# Comparison of Pilot-Scale Capacitive Deionization (MCDI) and Low-Pressure Reverse Osmosis (LPRO) for PV-Powered Brackish Water Desalination in Morocco for Irrigation of Argan Trees

**DOI:** 10.3390/membranes13070668

**Published:** 2023-07-14

**Authors:** Edgardo E. Cañas Kurz, Ulrich Hellriegel, Abdelkarim Hdoufane, Ibtissame Benaceur, Makram Anane, Fatima Jaiti, Abdelilah El-Abbassi, Jan Hoinkis

**Affiliations:** 1 Center of Applied Research, Karlsruhe University of Applied Sciences, 76133 Karlsruhe, Germany; edgardo.canas_kurz@h-ka.de (E.E.C.K.);; 2 Faculty of Sciences Semlalia, Cadi Ayyad University, Marrakech 40010, Morocco; 3 Faculty of Sciences and Technologies Errachidia, Moulay Ismail University, Errachidia 52000, Morocco; 4 Centre de Recherches et des Technologies des Eaux, Soliman 8020, Tunisia

**Keywords:** desalination, reverse osmosis, membrane capacitive deionization, agriculture, solar energy, sodium adsorption ratio

## Abstract

The use of saline water resources in agriculture is becoming a common practice in semi-arid and arid regions such as the Mediterranean. In the SmaCuMed project, the desalination of brackish groundwater (TDS = 2.8 g/L) for the irrigation of Argan trees in Essaouira, Morocco, to 2 g/L and 1 g/L (33% and 66% salt removal, respectively) using low-pressure reverse osmosis (LPRO) (*p* < 6 bar) and membrane capacitive deionization (MCDI) was tested at pilot scale. MCDI showed 40–70% lower specific energy consumption (SEC) and 10–20% higher water recovery; however, the throughput of LPRO (2.9 m^3^/h) was up to 1.5 times higher than that of MCDI. In addition, both technologies were successfully powered by PV solar energy with total water costs ranging from EUR 0.82 to EUR 1.34 per m^3^. In addition, the water quality in terms of sodium adsorption ratio was slightly higher with LPRO resulting in higher concentrations of Ca^2+^ and Mg^2+^, due to blending with feed water. In order to evaluate both technologies, additional criteria such as investment and specific water costs, operability and brine disposal have to be considered.

## 1. Introduction

The salinization of freshwater resources and changes in seasonal precipitation due to climate change are a serious and ongoing problem for the agricultural sector, mostly in arid and semi-arid regions. In the Mediterranean region, the decrease in precipitation has been estimated as 15–20% in recent years, whereas temperatures are estimated to be rising between 1 and 3 °C [1]. Another major factor limiting agricultural expansion in Mediterranean countries is the diminishing quality of water, e.g., through salinization. In North African countries, higher temperatures are linked to geographical location, leading to a higher evaporation in comparison with European and Middle Eastern regions; this is one of the most important factors increasing the groundwater’s salinity, next to seawater intrusion and geohydrological conditions [2,3,4]. Additionally, soil salinity is the second major cause of land degradation after soil erosion and has been a cause of agricultural decline for centuries, causing yield decrease for many crops and leading to desertification [5]. However, there is increasing pressure to use saline water to intensify agriculture produce, particularly in the most arid regions due to the lack of freshwater availability (low rainfall, limited groundwater supply). Therefore, adaptation strategies are needed to mitigate the effects of climate change on the water resources in the most affected regions, such as the implementation of high-end technologies like desalination processes and IoT (e.g., measuring soil moisture and plant water stress, drones or satellite imagery, etc.), as well as better agricultural management (such as adequate crop selection and deficit irrigation strategies) [6,7].

The joint EU-PRIMA research project “SmaCuMed” [8] aims at developing and testing an all-in-one smart irrigation cube system at pilot scale for smart-sensor controlled irrigation and the energy-autonomous treatment of brackish groundwater in remote agricultural Mediterranean regions. This study focused on the testing and comparison of low-pressure reverse osmosis (LPRO) with membrane capacitive deionization (MCDI)—for the first time at pilot scale—for the desalination of water to irrigate argan trees (*Argania spinosa*) in Morocco.

Argan is an endemic species of Morocco that has adapted genetically to persist under arid climates with highly variable annual rainfall and increased soil salinization, which typically makes it a robust solution against desertification and land degradation [9]. However, studies have shown that salt stress significantly affects the overall growing parameters of argan [10]. There has also been a significant reduction in the natural distribution of argan trees, with the most exposed and vulnerable trees disappearing due to severe drought. The low rate of soil regeneration due to the use of inappropriate farming methods and overgrazing has exacerbated the situation [11].

LPRO is a well-established membrane desalination process based on the principle of reverse osmosis (RO), which is considered to be the most optimized membrane-based desalination process today [12,13]. However, RO recovery rates are usually below 40% (for seawater). The LPRO process therefore aims to desalinate low-salinity brackish water (TDS: 1–15 g/L) using high-permeability membranes (e.g., [14]) operating at lower pressures (<16 bar) (hence its name), resulting in high flux and lower energy requirements [15,16,17,18]. Additionally, (LP)RO plants powered by renewable energy such as photovoltaic (PV) panels are becoming increasingly important at the community scale in remote places [19], with water prices ranging between 0.8 and 2.1 USD/m^3^ based on the type of energy supply [20].

MCDI is a rather new desalination process based on the electrosorption of salts onto porous carbon electrodes. By applying an electrical potential to the electrode pairs, the dissolved anions and cations are attracted by the oppositely charged electrode and are then stored in the pores of the electrodes in electrical double layers [21,22]. Once the electrodes are saturated, they can be regenerated by reversing the current or short-circuiting the electrodes (zero-current discharge). The main advantages over LPRO are the variable rejection rate, energy efficiency, and high water recovery [23,24]. However, the main challenges of MCDI are its low salinity ranges and higher investment costs, which are the main reasons why an application of MCDI on a large and industrial scale has not yet been achieved [22,24]. Studies have shown the long-term use of MCDI in different applications [25,26,27,28]. However, operation at larger operational scale is necessary to validate lab-scale and small-scale results and to better understand the effect of operational parameter interactions.

To date, only a few publications on pilot-scaled MCDI can be found wherein desalination was tested in an industrial environment. A study using MCDI modules to treat domestic wastewater [29] found the feasibility of reducing TDS concentration from ca. 0.7 g/L to < 0.25 g/L, with a total energy requirement of 1.28 kWh/m^3^. This value is in range of similar operations with RO listed in the same study. Another study showed the integration of MCDI in a photovoltaic energy supply system for the desalination of NaCl concentrations from 4 to 1–3 g/L, producing 2.3–5.23 m^3^/day and using 0.7–1.1 kWh/m^3^. In their study, they compared the energy values of a similar RO operation, which ranged from 0.93 to 4.3 kWh/m^3^ [30].

Another study compared the use of reverse osmosis with MCDI and LPRO in a modular system for the desalination of brackish river water (17 g/L) in Vietnam to produce drinking water, showing that both pilot plants resulted in similar energy requirements (ca. 5 kWh/m^3^) [28]. In the field of agriculture, one study evaluated the feasibility of MCDI in the desalination of brackish groundwater for the irrigation of selected crops at prices below AUD 1/m^3^, showing the profitability compared to irrigation with brackish water [31].

A report highlighted the economic advantages of different brine disposal strategies and the effect of feed water quality on traditional CDI compared to RO for feed salt concentrations < 5 g/L [32]. This paper showed that increasing the recovery rate (up to 54%) by adjusting the operating parameters of the CDI opens up different options for brine disposal and could reduce desalination costs by up to 36%.

Theoretical considerations comparing CDI and RO have been discussed only in a few publications [33,34,35]. Here, CDI models are compared with theoretical analysis of RO operations. Refs. [33,35] used theoretical models to show that RO outperforms CDI under most conditions in terms of energy consumption and thermodynamic efficiency. However, ref. [34] contradicted these results with experimental laboratory tests.

A direct comparison of RO and CDI at pilot-scale in the same environment (i.e., irrigation for agriculture) with feed salt concentrations above 2 g/L, has not been reported in peer-reviewed publications to date.

This paper presents and discusses for the first time the results of two desalination technologies, LPRO and MCDI for irrigation, during a one-year pilot project. In terms of applied research, this paper focuses on evaluating the applicability and operability of the systems at large scale, in a real environment, and over the long term. It aims to validate laboratory-scale results to provide a benchmark for parameters such as specific energy demand and water yield.

This paper does not include the results on argan growth and crop quality. The impact of water quality on argan crops will be published in a separate paper.

## 2. Materials and Methods

### 2.1. Pilot Site

A natural argan forest site located in Douar Foloust (31°23′43.8″ N 9°43′05.2″ W) in the south of Essaouira was chosen for the pilot trials. The Essaouira region is characterized by a semi-arid climate, where salinization and alkalization are the main risks that can affect the water used for irrigation [36]. A detailed physicochemical analysis of the water quality of samples taken from the pilot site between 24 December 2020 and 17 October 2022 is presented in Table 1.

### 2.2. Study Design

The water treatment units MCDI and LPRO were integrated in a Smart Cube as a holistic technological solution. The Smart Cube was designed for the desalination of groundwater and for smart irrigation control to desalinate both low (total dissolved solids, TDS < 3 g/L) and high salinities (3 < TDS < 10 g/L) in a modular design and to produce irrigation water with different qualities (i.e., salinities).

In this study, both MCDI and LPRO technologies were used in parallel to desalinate groundwater with a salinity of TDS = 2.8 g/L. The trials consisted of irrigating four different test plots of 20 argan trees each with different water qualities for a period of 341 days starting from 25 October 2021.

A detailed overview of the test plots and the experimental design is summarized in Table 2. The green field was irrigated using water directly from the well (2.8 g/L), whereas MCDI and LPRO were used for the irrigation of the yellow and blue plots. In the second half of the pilot tests, desalination targets of 33% and 66% were alternated to compare the two technologies. A fourth plot (red) was used as a control, which was only irrigated by rainfall. The amount of rainfall was monitored with a weather station and soil probes for soil moisture.

### 2.3. Plant Scheme

**Figure 1:**
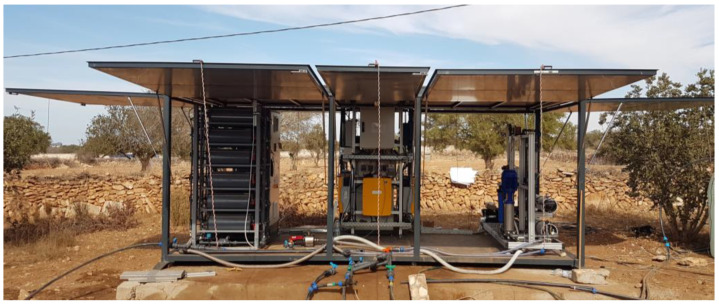
Smart Cube at pilot site of Essaouira: MCDI (**left**), power supply system (**center**), LPRO (**right**).

**Figure 2:**
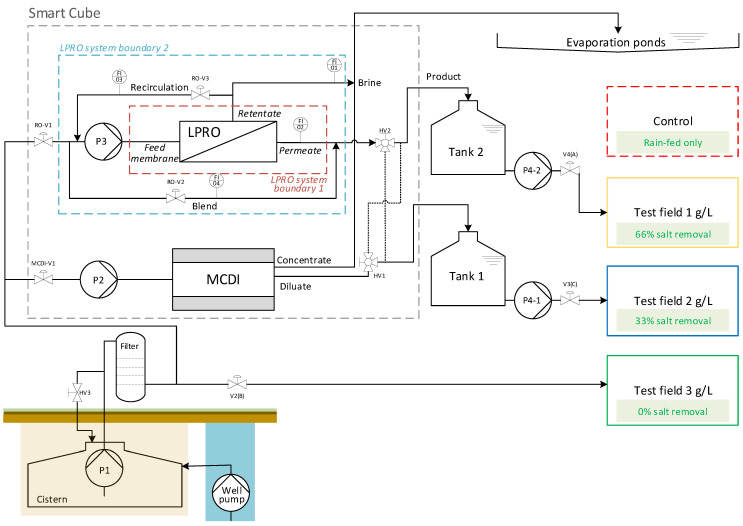
SmaCuMed plant scheme and study design—pilot site of Essaouira.

The Smart Cube and the concept scheme of the pilot plant are shown in Figure 1 and Figure 2, respectively.

Desalination: Brackish groundwater from the well was pumped using a submersible pump (Lowara 4Gs22M-L4C, 2.2 kW, GENVIK 4KP) and stored in an underground cistern tank (volume ca. 30 m^3^). A cistern pump P1 (GENVIK 4KP-4-11, 1.1 kW, Villankurichi, India) delivered the brackish water through a pre-filter (microfilter 1 µm, replaced later by a sand filter with one-third sand and gravel plus two-thirds BIRM^®^ material for iron removal, Postbauer-Heng, Germany) to the desalination systems in the Smart Cube. The LPRO (Schaller I.W. GmbH, RO-PVCU2000, Meckesheim, Germany) consisted of two low-fouling spiral wound membrane modules made of composite polyamide (Nitto ESPA2-LD, Osaka, Japan) with a pressure pump P3 (KSB Movitec VSF006-16, 4 kW, Frankenthal, Germany) to operate at pressures p_max_ = 15 bar. The MCDI (Voltea Inc. IS12-H, Dallas, TX, USA) system consisted of 12 electrode modules (Voltea Inc. C18) with 18 × 24 electrode pairs (14 cm × 14 cm) each. The system pump P2 (Grundfos, CM10-3, 2.2 kW, Bjerringbro, Denmark) was only used for cleaning the MCDI system. Both desalination systems had a cartridge filter (1 µm) for particle filtration.

Irrigation: Treated water (TDS = 1 and 2 g/L) was stored in storage tanks 1 and 2 (V = 3 m^3^) for the irrigation of blue and yellow fields, respectively. Irrigation was carried out using delivery pumps (P4-1 and P4-2) (LEO, XCm170-1). The green field (untreated feed water, TDS ≈ 3 g/L) was irrigated directly from the cistern using cistern pump P1 and automatic valve V2.

Brine treatment: The concentrate stream (waste) generated during the desalination processes (regeneration phase during MCDI; continuous during LPRO) was discharged to a catchment basin (evaporation pond, A = 810 m^2^) covered with an impermeable geo-membrane in order to avoid leaching of the brine into the soil and groundwater. Through the evaporation process, salt deposits were produced in the basins, which needed to be disposed of separately.

Energy supply: The Smart Cube was powered by 19 foldable solar PV-panels (Suntech Power STP405S-A72, Wuxi, China) with an output of 7.7 kWp on a total area of 38.2 m^2^ and a battery system consisting of 6 lithium batteries (BYD B-Box Premium LVS 24.0, Shenzhen, China, lithium iron phosphate-LFP) with a total capacity of 24 kWh, 1 inverter (Sunny TriPower 6.0-1AV, Kassel, Germany), and 3 battery inverters (SunnyIsland 6.0H).

Controlling: The system was automatically operated by a Siemens LOGO! PLC (LOGO! 12/24RCE Version 8.3, with 1 LOGO! DM16 24R and 2 LOGO! AM2 expansion modules).

Cleaning and maintenance: The desalination plants were cleaned periodically (2–3 times during the testing period) with citric acid for scaling mitigation. Additionally, the MCDI apparatus was also cleaned using an NaOCl solution and an air–water mixture (air scour/mechanical cleaning) for fouling mitigation. The LPRO cleaning was carried out manually. MCDI cleaning was programable via the HMI and ran semi-automatically. The cleaning solutions were prepared in separate tanks.

### 2.4. LPRO Process

The LPRO desalination plant consisted of two ESPA2-LD 8040 membranes in series. As shown in Figure 2, the LPRO process was characterized based on two system boundaries.

System boundary 1 only included the flows directly into and out of the membrane module and was used to characterize membrane efficiency (membrane feed, permeate, retentate). System boundary 2 included recirculation and mixing for overall process evaluation (feed, product, brine). All necessary volume flows (Q_brine_, Q_permeate_, Q_recirculation_ and Q_blend_) were measured with variable area flowmeters (GEMÜ 800).

### 2.5. MCDI Process

The MCDI process was operated in constant current mode using four power supplies in parallel for 3 electrode modules in series. The operation included the following phases:

1. Charge phase for desalination/adsorption at constant current;
2. Discharge phase for regeneration of the electrodes at reversed constant current (waste stream);
3. Pre-charge phase at constant current used to flush the waste stream out of the modules before the desalination phase;
4. Shunt phase before and after the discharge phase for electrically discharging the electrodes by short-circuiting the electrodes. This was done in order to balance the electrical potential between the electrodes without using any energy input and only by using the potential difference between electrodes (no use of external current/zero-current).

The MCDI parameters are listed in Table 3.

### 2.6. Data Evaluation

The salt removal or rejection rate (RR), which yields the ratio between the salinity of the product and the feed, indicates the degree of desalination in percent. The salinity *S* (or total dissolved solids, TDS in g/L) was calculated by measuring the conductivity σ (µS/cm) and using a conversion factor of *k* = 0.64 based on the literature [37].

(1) $$RR=\frac{\sigma_{feed\;}-\sigma_{prod}}{\sigma_{feed\;}}\cdot 100\%$$

$\sigma_{feed}$ = conductivity of feed (groundwater) in μS/cm
$\sigma_{prod}$ = conductivity of product (LPRO) or diluate (MCDI) in μS/cm

The water recovery (WR) indicates the ratio of the resulting product volume and the volume of the feed water in percent. Desalination processes with high water recovery make an optimal use of the resource. High water recoveries are preferred, especially in regions that suffer greatly from water stress or water shortages.

(2) $$WR=\frac{V_{prod}}{V_{feed\;}}\cdot 100\%$$

$V_{prod}$ = total volume of produced product (LPRO) or diluate (MCDI) in m^3^
$V_{feed}$ = feed volume in m^3^

Additionally, the results of RR and WR for LPRO can be calculated for the membrane depending on system boundary 1 (membrane), as shown in Figure 2. From Equations (1) and (2), this results in:

(3) $${RR}_{membr}=\frac{{\sigma_{feed}-\sigma }_{permeate}}{\sigma_{feed\;}}\cdot 100\%$$

and

(4) $${WR}_{membr}=\frac{V_{permeate}}{V_{feed\;}-V_{blend}+V_{recirculation}}\cdot 100\%$$

The specific energy consumption (SEC) is the energy required to produce one cubic meter of desalinated product. The higher the SEC value, the more energy intensive the desalination process. The SEC is calculated as:

(5) $${SEC}_{system}=\frac{E_{system}}{V_{prod}}$$

$E_{system}$ = energy consumption of system (MCDI or LPRO) in kWh
$V_{prod}$ = volume of product water (LPRO) or diluate (MCDI) in m^3^
for which $E_{system}$ is estimated as:
$E_{LPRO}$ = average performance of LPRO recorded during the desalination process (retrieved online via SunnyPortal, ennexOS) in kWh
$E_{MCDI}$ = energy meter reading at end of each test in kWh

The energy meter of MCDI monitored only the energy consumed by the MCDI system without the feed pump (P1). The cistern pump (P1) was used as a feed pump for both desalination systems. However, it was designed for the operation of the LPRO system, which required higher volume flows and pressures than necessary for MCDI. Therefore, the MCDI feed pressure was regulated with a recirculation flow using a hand valve after P1 (see Figure 2).

Pump power (P1) for MCDI was calculated theoretically as follows:

(6) $$P=\frac{\;p\;\cdot \;Q}{\eta }$$

*P* = power in watts
*p* = operating pressure in Pa
*Q* = volume flow in m^3^/s
η = pump efficiency (80–95%)

Additionally, the charge efficiency of the MCDI system [38], which is the ratio of adsorbed salts over charge (both expressed in mol), was calculated for the charge phase as follows:

(7) $$𝛬=\frac{\left(c_{0}-C\right)\cdot \;VF\;}{\int Idt\cdot \;M}$$

𝛬 = charge efficiency (mol/mol)
*C*_0_ = initial concentration in g/L
*C* = residual concentration in g/L
*V* = total phase volume in L
*F* = Faraday constant in C/mol
*I* = average applied current in A*
*t* = phase time in s
*M* = molar mass in g/mol

* Current was not logged by the system; however, set currents were assumed to be constant for the entire charge cycle since the maximum voltage for charging the modules was never reached.

Furthermore, the sodium adsorption ratio (SAR) and soil sodicity (%Na) were calculated for the evaluation of irrigation water quality. Sodium contributes directly to the total salinity and may also be toxic to sensitive crops, including fruit trees like argan. Long-term irrigation with low-quality, highly saline–sodic water may increase soil salinity and cause irreversible soil structural degradation, reducing the soil water movement or saturated hydraulic conductivity (Ks), which describes its ability to transmit water in a saturated condition [39]. The presence of divalent cations Ca^2+^ and Mg^2+^ in irrigation water can reduce the risk of soil sodicity (excess of Na^+^). SAR is calculated as the ratio of the concentration of monovalent Na^+^ and divalent cations Ca^2+^ and Mg^2+^ and can be used for estimating the water permeability of soils [5].

(8) $$SAR=\frac{{Na}^{+}}{\sqrt{\frac{{Ca}^{2+}+{Mg}^{2+}}{2}}}$$

and

(9) $$\%Na=\frac{{Na}^{+}+K^{+}}{{Ca}^{2+}+{Mg}^{2+}{Na}^{+}+K^{+}}\times 100\%$$

with concentrations of Na^+^, K^+^, Ca^2+^, and Mg^2+^ in mmol/L.

### 2.7. Analytical Method

Monitoring: Experimental data were measured on site (WTW TetraCon 325/Pt, Hanna HI98129/130; pH: WTW pH 325) and monitored online. IoT sensors located on the respective irrigation lines (green, blue, and yellow lines) were used for monitoring the flow rate and conductivity. These data were accessed via an online portal (www.myirrigation.com, accessed on 23 May 2023). Energy consumption and uptake of the Smart Cube were retrieved and analyzed via the online platform ennex OS Sunny Portal by SMA (www.ennexos.sunnyportal.com, accessed on 23 May 2023).

Analysis: Selected samples were collected and sent to Germany for ion chromatography (Metrohm AG, 883 Basic IC plus, Herisau, Switzerland) and measurement of total organic content (Shimadzu, TOC-L, Tokyo, Japan).

## 3. Results

### 3.1. Desalination Performance

#### 3.1.1. LPRO vs. MCDI

**Figure 3:**
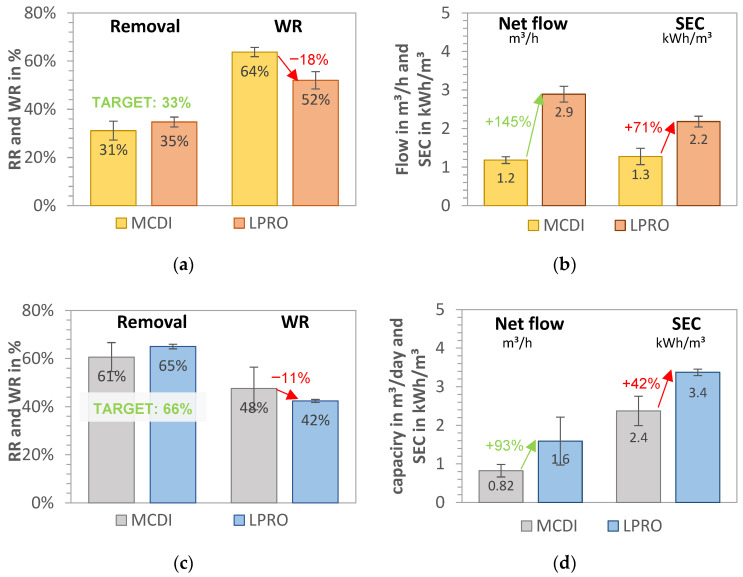
MCDI vs. LPRO—comparison of WR, net flow, and SEC for set removals of (**a**,**b**) RR = 33% (yellow field, TDS_product_ = 2 g/L) and (**c**,**d**) RR = 66% (blue field, TDS_product_ = 1 g/L). Feed: 2.8 g/L.

A comparison of the two desalination processes (MCDI and LPRO) for the salt removal targets of 33% and 66% is shown in Figure 3a–d for all data collected during the pilot tests (341 days).

Water recovery of the LPRO system (system boundary 2) was achieved at WR_LPRO,33%_ = 52.0% ± 3.6% and WR_LPRO,66%_ = 42.4% ± 0.6% for the 33% and 66% targets, respectively. In terms of energy demand, the SEC increased from SEC_LPRO,33%_ = 2.18 ± 0.14 kWh/m^3^ to SEC_LPRO,66%_ = 3.37 ± 0.08 kWh/m^3^, representing a 35% increase in energy demand for the desalination from 2.8 g/L to 1.8 and 1.0 g/L, respectively. By doubling the RR (33% to 66%), the total daily production of irrigation water decreased by 80%, with the throughput falling from 2.90 to 1.60 m^3^/h (net product flow).

The MCDI system had a water recovery of WR_MCDI,33%_ = 63.7% ± 3.4% and WR_MCDI,66%_ = 47.6% ± 8.8% for the 33% and 66% salt removal targets, respectively. By changing the operational parameters for increased removal (RR = 66%), the throughput was reduced by 30%, from 1.18 m^3^ product water/h to 0.82 m^3^/h. The results of the SEC_total_ of MCDI process (including MCDI and theoretical pump consumption) were calculated as SEC_MCDI,33%_ = 1.3 kWh/m^3^ ± 0.2 kWh/m^3^ and SEC_MCDI,66%_ = 2.4 kWh/m^3^ ± 0.5 kWh/m^3^, showing that almost double the energy capacity was needed for the removal of an additional 1 g/L salt (e.g., doubling the removal target of 33% to 66%). A comparison of the SEC of the pump (measured on site) was theoretically calculated, revealing that the measured values were up to 1.5 kWh/m^3^ higher and that up to 54% energy could be saved in the MCDI desalination by dimensioning the feed pump accordingly.

In summary, the results for 33% target removal in Figure 3a,b show that MCDI had 10–20% higher water recovery, which was about 70% lower for MCDI with SEC than LPRO. However, the throughput productivity of LPRO was more than double that of MCDI, with a net flow of Q_LPRO_ = 2.9 m^3^/day.

By increasing the target removal from 33% to 66% (Figure 3c,d), the WR and the daily net flows were reduced by about 20%. MCDI showed better WR and SEC values, with 11% higher WR and 38% higher SEC, whereas LPRO showed more than double the capacity. These results demonstrate that the differences in MCDI and LPRO were less pronounced for the higher removal target (66%), which indicate that MCDI’s advantages in terms of WR and SEC are greater for lower rejection rates and that it is therefore better in applications with low salinities or lower salt removal targets. However, there was a slight difference in the removal RR achieved for both systems on average (31% vs. 35% and 61% vs. 65%, respectively), which may have had an influence on the total results.

#### 3.1.2. Effect of RR and WR on SEC

**Figure 4:**
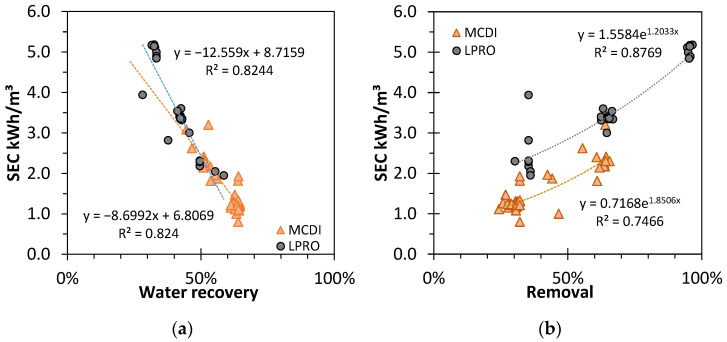
Effect of (**a**) removal rate (RR) and (**b**) water recovery (WR) on the specific energy consumption (SEC) for different results of MCDI and LPRO. TDS: feed: 2.8 g/L.

Throughout the trials, different process setting parameters for MCDI (cycle time, phase time, applied currents and voltages, flows, shunt times, etc.) and LPRO (recirculation, blending, operating pressures, etc.) were used to achieve the set target desalination rates. In order to provide a broader look at the relationship between RR, WR, and SEC, the results for all desalination tests carried out throughout the trials were determined for both MCDI and LPRO and are displayed in Figure 4a,b. Even with a complex relationship between the parameters set, a general trend was observed: Higher water recoveries and lower removal rates resulted in lower SEC values.

The SEC decreased linearly with higher WR for both LPRO and MCDI (R^2^ = 0.82). The intersection point for both trendlines at WR ≈ 50% indicates the importance of yield for the total performance efficiency of the processes; with a steeper decrease, the influence of WR in LPRO was more pronounced.

In contrast, with higher salt removal rates, the SEC increased exponentially (R^2^ = 0.83 and R^2^ = 0.72, respectively), and LPRO needed approx. +Δ0.7–0.9 kWh/m^3^ more energy than MCDI in these settings.

Overall, these results prove that MCDI is more energy efficient and environmentally friendly in terms of resource efficiency and energy demand than LPRO, mostly due to the lower desalination rates, whereas the membrane system showcased a larger capacity and output productivity.

#### 3.1.3. Effect of Volume Flow and Pre-Pressure

In order to study the effect of different parameters on the desalination processes at pilot scale, tests were carried out using different feed pressures and volume flows. The feed pressure was regulated with a recirculation flow using a hand valve after the cistern pump. The same pump setting for P1 was used for both desalination technologies.

**Figure 5:**
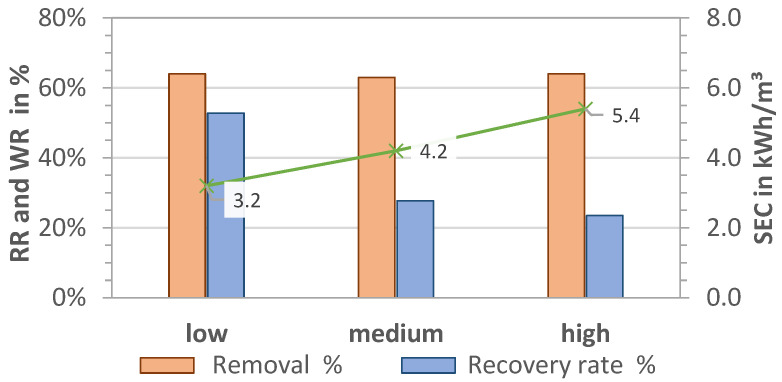
Effect of pre-pressure on removal (RR), water recovery (WR), and SEC in MCDI. TDS feed: 2.8 g/L; Q_feed_ = 27.9, 28.7, 29.7 L/min.

**Figure 6:**
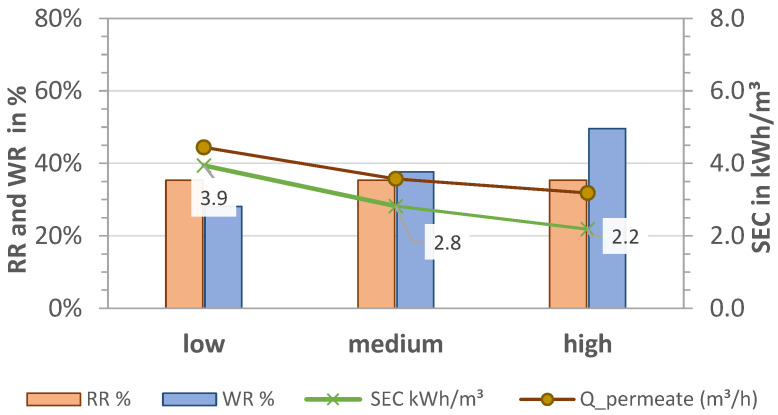
Effect of pre-pressure on removal (RR), water recovery (WR and WR_membrane_), and SEC in LPRO. TDS feed: 2.8 g/L; Q_feed_ = 4.9, 5.5, 5.6 m^3^/h.

The results for RR, WR, and SEC for pre-pressures p_low_ = 1.7 bar, p_medium_ = 2.1 bar, and p_high_ = 2.5 bar (P1) and thus different volume flows are shown in Figure 5 and Figure 6 for MCDI and LPRO, respectively. The data show that higher flows had an overall negative effect on all parameters of MCDI for an equal rejection rate (RR = 64%) by increasing the SEC from 3.2 to 5.4 kWh/m^3^ (41% increase) and reducing the WR by half, from 53% to 24%.

In contrast to MCDI, higher pre-pressures had a positive effect on LPRO, increasing the recovery rates from 28% to 50% and reducing the overall energy consumption by almost half, from 3.9 to 2.2 kWh/m^3^. This is because higher feed flows resulted in higher permeate flows (0.5–1.0 m^3^/h). In order to maintain a constant product quality at 2 g/L, the blend flow (bypass) was increased from 870 L/h to 1300 L/h and 1750 L/h while adjusting the concentration recirculation flow. Additionally, with the increase in the WR rate, the energy consumption of the process was improved in accordance with Figure 4b, resulting in SEC = 2.2 kWh/m^3^.

### 3.2. LPRO—Flux and Permeability

**Figure 7:**
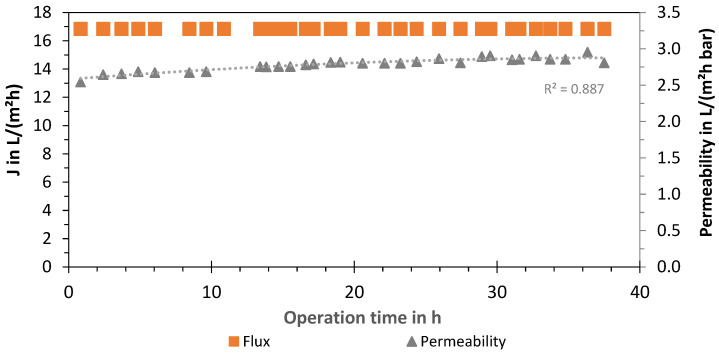
Flux and permeability of LPRO membrane (system boundary 1) over time (cumulative operation time: 320 days).

Additionally, an evaluation of the membrane of the LPRO system (2 times Nitto, ESPA-LD 8040) was carried out by analyzing the permeate flux *J* and permeability of the process (system boundary 1). The results for the experiments with 66% removal are presented in Figure 7, showing a constant flux at *J*_66%_ = 16.8 L/(m^2^ h) with a membrane area of 37.1 m^2^ per module. The LPRO was operated at a constant permeate flow of 1250 L/h by adjusting the concentrate volume flow (2500 L/h) and feed pressure (6.2 ± 0.5 bar). The average permeability was measured as 2.8 ± 0.1 L/(m^2^ h bar) and the membrane recovery was WR_membr,66%_ = 33.4% ± 0.5%. Although no increase in the feed pressure over time was needed, the permeate quality increased from 130 to 200 µS/cm with an average conductivity of 174 µS/cm. Product conductivity was regulated by blending with the feed using the bypass valve (580 L/h).

For 33% removal experiments, the membrane flux and permeability were *J*_33%_ = 13.5 ± 1.3 L/(m^2^ h) and 2.5 ± 0.4 L/(m^2^ h bar), respectively. However, the blend was increased to 1750–1900 L/h to adjust the product conductivity, and increased feed flows were needed so that the recirculation valve was open to 2000 L/h. Therefore, the membrane recovery rate decreased to WR_membr,33%_ = 26% in comparison to the experiments with 66% removal. An additional experiment was carried out to test the membrane at the same recovery rates. By closing the recirculating valve, water recovery was increased to 36%, whereas the flux and permeability achieved were 17.9 L/(m^2^ h) and 2.8 L/(m^2^ h bar), respectively.

Additionally, LPRO was evaluated for the production of permeate (without blending). The results are summarized in Table 4, which shows an RR_membr_ = 95.4% for a concentration of 120 mg/L and a WR_membr_ = 32.7%. The flux and permeability were on average 16.5 L/(m^2^ h) and 2.4 L/(m^2^ h bar), respectively with a membrane active area of two membrane modules of A_m_ = 74.4 m^2^ (average feed pressure of 6.2 ± 0.5 bar). The WR_membr_ was kept < 33%, as recommended by the manufacturer, so that the beta value of a maximum of 1.2 (concentration polarization above 20% at the membrane compared to the bulk) was not exceeded [40]. This conservative operation approach favors a lower scaling potential (“low-recovery–low-scaling”) and avoids dosing of antiscalant.

### 3.3. MCDI—Current Density and Charge Efficiency

The MCDI was operated at a constant current with the applied voltages ranging from 0.63 to 0.70 V and from 0.67 to 1.13 V per module for the 33% and 66% salt removal targets, respectively. The system was operated with an average current density during the charge and discharge phases of 8.2 A/m^2^ and 12.9 A/m^2^ for the 33% target, and 18.4 A/m^2^ and 18.6 A/m^2^ for the 66% rejection target. These values are in agreement with the values found in the literature of 5–20 A/m^2^ and an operation with lower voltages to avoid Faradic reactions in the electrode surface [41,42]. The increased values during the discharge phase are directly linked to the higher currents chosen for the regeneration phase (high current, short phase time, less waste).

The charge efficiency yields the ratio between electrical charge (power supply) and ionic charge (removed salts). The charge efficiency of the electrode modules during both phases (33% and 66% removal) was calculated as *Λ*_33%_ = 77.1% and *Λ*_66%_ = 61.2%, respectively. Although the applied current needed for RR = 66% (185 A) was more than double R = 33% (80 A), the resulting electrical charges were almost the same due to the shorter cycle times (see Table 2). On the other hand, the total number of effective ions removed in a cycle time was much lower, indicating that the difference in net volume flow had a major effect on the effective removal efficiency. The decrease in *Λ* with higher salt removal targets may be related to two effects: the higher electrical resistances when running at higher currents and the higher voltages, which may have led to higher faradaic reactions, hindering the electrode. In addition, the cable thickness of the electrodes in the IS12 was one factor that could be improved in relation to the higher currents in order to lower resistance and operate safely at the applied currents.

### 3.4. Specific Ion Removal

**Figure 8:**
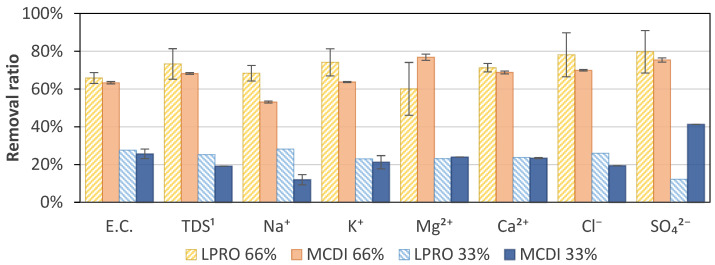
Specific ion adsorption at 66% and 33% removal for MCDI and LPRO. ^1^ TDS feed: 2.8 g/L.

The results of the specific ion removal of LPRO and MCDI for the targets of 33% (TDS ≈ 1.7–1.9 g/L) and 66% (TDS ≈ 0.8–0.9 g/L) salt removal are shown in Figure 8. At a target removal of 33%, the analysis showed a difference in both technologies, with concentrations of Na^+^ of 294 mg/L and 355 mg/L and Cl^−^ of 1.2 g/L and 0.69 g/L for MCDI and LPRO, respectively. When increasing the target to 66%, the values decreased to concentrations of Na^+^ of 136 mg/L and 192 mg/L and Cl^−^ of 0.34 g/L and 0.49 g/L, respectively. This showed that there was a higher difference in removal between 33% and 66% removal with MCDI than LPRO, with MCDI having notably higher removals of Cl^−^ (70%) than Na^+^ (53%) at 66% removal.

However, it was not possible to identify a general trend, and the specific removal should be evaluated individually depending on the desired requirements. In general, the results show that divalent ions (Ca^2+^, Mg^2+^, and SO_4_^2−^) tended to be removed to a greater extent than monovalent ions (Na^+^ and K^+^). The specific absorption rate of different ions also varied with ion size, hydration radius, and charge [43,44,45,46]. Yet, a time dependence and an effect of the initial specific ion concentration in the bulk solution can affect the specific removal and selectivity [44,47], as in the example of Cl^−^, which showed a slightly higher specific removal than the average removal. However, no differences were observed for the two rejection rates.

**Figure 9:**
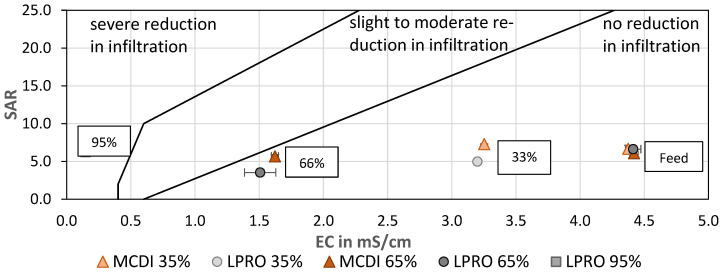
SAR vs. EC—effect of desalination on irrigation water quality for soil infiltration. TDS: feed: 2.8 g/L (mod. from [39]).

In order to evaluate the quality of the treated water for irrigation, the sodium adsorption ratio (SAR) was calculated and evaluated as a function of the EC (Figure 9). SAR is used to assess infiltration problems due to an excess of sodium in relation to calcium and magnesium, since increased Na^+^ levels are associated with degradation of soil structure and a decrease in soil infiltration and permeability [48,49]. Although the SAR of the irrigation water remained almost constant after treatment by MCDI and LPRO, the 66% reduction in EC shifted the SAR:EC ratio to the border of the low-to-moderate risk area in the SAR:EC diagram, which should be considered when using water for irrigation. In general, the results showed that the SAR values of LPRO were slightly lower than those of MCDI and therefore remained in the zone where no reduction in infiltration is observed This is due to the blending of permeate water with feed water, since LPRO removes a high degree of bivalent ions.

In addition, the SAR value of the LPRO permeate (RR_membr._ = 95%) was evaluated and compared with the irrigation water (66% and 33%), showing that irrigation with permeate could cause a severe reduction in the soil infiltration quality, highlighting the effect of desalination and the importance of the salinity ratio for the water quality (Figure 9).

### 3.5. Brine Disposal

Concentrate or brine disposal in evaporation ponds was used in the pilot trials to protect soil and groundwater from salinization through leaching of brine salts and thereby reducing the environmental impact [50]. In addition, evaporation ponds are relatively easy to construct and require little maintenance and operator attention compared to mechanical systems [51]. Other brine disposal methods are deep-well injection, seawater mixing, and horticulture with salt-tolerant plants (e.g., halophytes) [13,52]. However, high-recovery (WR) operations with minimal waste disposal (zero-liquid discharge) are strongly encouraged, especially in arid regions, to minimize water loss and integrate an effective water usage [32].

During the one-year trials, approximately 145 m^3^ of concentrate was discharged into the evaporation basins, corresponding to a total of approx. 550 kg of salt. Salts obtained from the basins could have potential industrial uses. However, two main problems were identified in the design: The evaporation ponds were open and impurities such as dirt and sand could have contaminated the salt. A sample of the salt was taken and found to contain 84% (*w/w*) salt, 15% (*w/w*) sand, and 1% (*w/w*) foliage. Secondly, the harvesting of salt from the ponds is not a common practice in the region, and therefore personnel training and capacity building is required, which increases operational costs and labor. In addition, given the low cost of salt, its use for industrial or commercial purposes cannot be recommended at this time. The acquired salt can be discharged into the sea without harming the environment.

Of the various brine disposal methods available, horticulture with halophytes is the most recommended and should be investigated further. This ensures that no brine is discharged into sensitive environments, and the halophytes can be used as a by-product, for example, as animal feed.

### 3.6. PV Energy Supply

**Figure 10:**
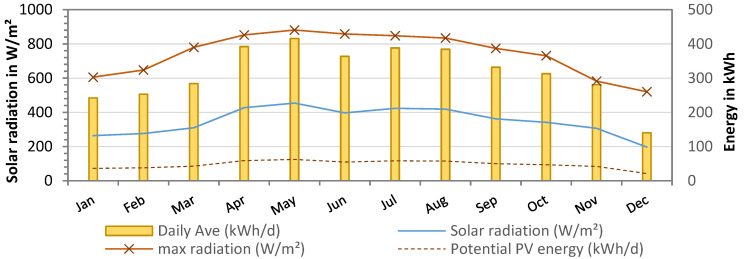
Solar radiation from weather data (W/m^2^) at pilot site (from January to December 2022) and theoretical PV energy supply in kWh (15% efficiency).

A summary of the solar radiation measured over the course of one year at the pilot site in Essaouira is shown in Figure 10. Solar radiation averaged 348 ±76 W/m^2^, with the highest values between May and August (maximum radiation: 880 W/m^2^). With a PV panel area of 38.2 m^2^, the available solar energy was calculated as 16,572 kWh per year or 316 ± 77 kWh daily. As the desalination was operated only two times per week, the power generation was not continuous. Therefore, the total energy requirement during the pilot tests from November 2021 to October 2022 was only 3596 kWh, with an average of 10 ± 2 kWh/day. This was due to the fact that the power supply of the PV was automatically adjusted to the energy consumption. The theoretical energy supply of the PV panels was calculated to be 47 ± 12 kWh/day, assuming a system efficiency of 15% (18% PV panels—3% system losses).

**Figure 11:**
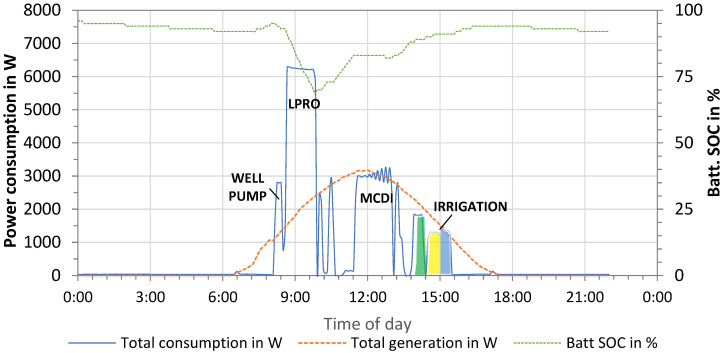
Energy supply and demand—monitoring of the PV system (February 2022).

Additionally, the PV energy supply and demand showing all operation processes in one day is presented in Figure 11. The PV power generation started with sunrise at around 07:10 and reached peak power of 3200 W at noon. The total energy produced by the PV system on this day was 19.2 kWh.

The energy demand was monitored and included the pumping of well water (“well pump”, P = 2.8 kW), LPRO for 66% removal (P = 6.24 kW), MCDI for 33% removal (P = 2.96 kW), and irrigation of the three fields (P_green_ = 1.8 kW; P_yellow_ = 1.3 kW; P_blue_ = 1.4 kW). The total energy consumption resulted in 13.59 kWh/day for the irrigation of three fields (E_irrigation_ = 1.70 kWh) and the production of 2 m^3^ irrigation water with LPRO (E_LPRO,66%_ = 7.25 kWh) and MCDI (E_MCDI,33%_ = 4.64 kWh) (1 m^3^ irrigation + 1 m^3^ reserve). Compared to the total supply, this corresponded to a surplus of energy of 5.57 kWh, which was used for battery charging, as illustrated by the dotted green line (state of charge, SOC).

Battery storage (E_total_ = 24 kWh) was used in different irrigation scenarios, wherein the fields were irrigated in the early morning and desalination started at noon (highest power production). However, both the PV and battery capacity were over-dimensioned for the tests, as irrigation was limited to only 1 m^3^ per field.

With the available PV energy of 47 kWh/day on site (see Figure 10) and the SEC results (see Figure 3a–d), the potential capacity of each technology (desalination only) was calculated as 4.2 and 7.8 m^3^/day for MCDI and 3.0 and 4.6 m^3^/day for LPRO for the salt removal targets of 33% and 66%.

## 4. Cost Estimation

A cost estimate for the Smart Cube (LPRO + MCDI + PV system) was carried out based on a 20-year period of use. Actual material and purchase costs were used. The water production (350 operating days per year) was based on a theoretical daily PV production of 47 kWh/day (see Section 3.5). A life span of 5 years was assumed for the replacement of membranes (2 for LPRO) and electrode modules (12 for MCDI), and 10 years for the pumps, battery system, and inverters. In addition, material and labor costs for maintenance and cleaning were calculated, including repair and replacement of spare parts (5%/year of initial cost) and chemicals (NaOCl, citric acid, NaOH, oil change, etc.). Labor was calculated at 1 h/week for MCDI, 0.5 h/week for LPRO, and 1 h/month for PV cleaning at an hourly rate of MAD 15.55 per hour (or EUR 1.55 per hour) [53]. The cost calculations did not include well pumping or concentrate disposal.

The calculations are presented in Table 5, which shows a total annual cost of CAPEX = EUR 2511 and EUR 2950 for LPRO and MCDI, respectively. The operating costs (OPEX) were calculated to be approximately EUR 550. In addition, replacement costs were calculated at EUR 2846 per year for LPRO and EUR 5870 for MCDI, which was significantly higher due to the number and actual price of the electrode modules. However, this price is expected to decrease in the future.

In total, specific water costs were calculated as 0.78 EUR/m^3^ for LPRO and 0.72 EUR/m^3^ for MCDI for the production of irrigation water with 2 g/L (salt removal of 33%). The lower prices for MCDI are directly related to the lower SEC and higher WR than for LPRO.

On the other hand, increasing the salt removal target to 66% increased the costs to 1.21 EUR/m^3^ and 1.34 EUR/m^3^ for LPRO and MCDI, respectively. The increase in water costs from EUR 0.72 to EUR 1.34 (85% increase) at higher rejection rates by using MCDI was attributed to the sharp decrease in water yield, so that the total specific water cost for 1 g/L was lower with the LPRO. This again highlights the better performance of the MCDI at lower salt removal rates.

## 5. Conclusions

- In this paper, two desalination systems for the production of irrigation water from brackish groundwater were compared at pilot scale in a real environment over the course of one year.
- The performance comparison of MCDI and LPRO showed the potential of MCDI coupled with PV as an environmentally friendly process with lower energy requirements and higher recovery rates than LPRO. Optimized MCDI operation was achieved by reducing the volume flow rates and feed pressure and by using a zero-current phase (shunt) prior to electrode regeneration to save energy.
- LPRO showed a higher net flow (throughput) and simpler operating requirements, which are essential to reduce specific water costs.
- Evaporation ponds were found to be easy to construct and feasible for brine disposal, but operation at higher water recovery to minimize brine production towards zero liquid discharge is still preferred.
- In order to evaluate both technologies for agricultural use, criteria other than water quality and plant capacity should be considered, such as investment and specific water costs, operability, and brine disposal options.
- The Smart Cube was designed to integrate both LPRO and MCDI. However, a future application may consider only one desalination technology.

## Figures and Tables

**Table 1:** Analysis of groundwater at pilot site of Essaouira, Douar Foloust (no. of samples: 12).

Parameter ^a,b^	Unit	Total Avg.	Std. Dev.
pH	-	7.5	±0.1
E.C.	mS/cm	4.4	±0.3
TDS ^c^	g/L	2.8	±0.1
TOC	mg/L	5.3	±6
HCO_3_^−^	mg/L	244	±41
Na^+^	mg/L	427	±52
K^+^	mg/L	12	±3
Ca^2+^	mg/L	198	±49
Mg^2+^	mg/L	232	±59
Cl^−^	mg/L	1543	±112
SO_4_^2^^−^	mg/L	102	±30
TDS (∑) ^d^	g/L	2.5	±0.2

^a^ Analysis with Metrohm AG, 883 Basic IC plus and Shimadzu TOC-L. ^b^ Samples taken from December 2020 to October 2022. ^c^ From E.C. value (*K* factor 0.64; [37]). ^d^ Sum of cations and anions.

**Table 2:** Overview of irrigation of pilot fields/plots.

Field	Target TDS (g/L)	Source	Salt Removal ^2^ (%)	No. of Argan Trees
Green	3.0 ^1^	Well	0%	20
Blue	2.0	MCDI/RO	33%	20
Yellow	1.0	RO/MCDI	66%	20
Red	Rainwater	Rainfall	N/A	20

^1^ Avg. salinity of groundwater after trials, TDS = 2.8 g/L. ^2^ Averaged values.

**Table 3:** Avg. MCDI settings for experiments with 33% and 66% removal (right values). Operation at constant current at max 1.2 V per electrode module.

Phase	Flow Rate (L/min)		Cycle Time (s)		Applied Current (A)	
	33%	66%	33%	66%	33%	66%
Discharge	30.9	30.1	145	50–130	130	180–200
Pre-charge ^1^	31.6	29.9	20	30–45	80	175–200
Charge	31.2	28.0	370/400	160–200	80	175–200
Shunt ^2^	30.9	30.1	50	55	Shunt	Shunt

^1^ In the pre-charge phase, water volume is discarded as brine. ^2^ Before the discharge phase (after discharge shunt time was fixed to t_shunt_ = 5 s).

**Table 4:** Avg. evaluation parameters of LPRO membrane (2 times Nitto, ESPA2-LD 8040).

TDS Feed g/L	TDS Permeate g/L	Rejection Rate RR_membr_ %	Water Recovery WR_membr_ %	Flux Membrane L/(m^2^ h)	Permeability L/(m^2^ h bar)
2.84	0.12	95.4 ± 0.6	32.7 ± 0.6	16.5 ± 0.5	2.4 ± 0.1

**Table 5:** Yearly costs (in EUR) and total water cost (in EUR/m^3^) of the Smart Cube using (a) LPRO and (b) MCDI for salt removal rates of RR = 33% and RR = 66%.

	(a) LPRO + PV	(b) MCDI + PV
CAPEX	2511	2950
OPEX	592	533
Replacement	2846	5870
Water cost per m^3^ (RR = 33%)	0.78	0.72
Water cost per m^3^ (RR = 66%)	1.21	1.34

## Data Availability

The data presented in this study are available on request from the corresponding author.
